# Leaders in Interdependent Contexts Suppress Nonverbal Assertiveness: A Multilevel Analysis of Japanese University Club Leaders' and Members' Rank Signaling

**DOI:** 10.3389/fpsyg.2018.00723

**Published:** 2018-05-31

**Authors:** Atsuki Ito, Matthias S. Gobel, Yukiko Uchida

**Affiliations:** ^1^Graduate School of Human and Environmental Studies, Kyoto University, Kyoto, Japan; ^2^Department of Psychological and Brain Sciences, Sage Center for the Study of the Mind, University of California, Santa Barbara, Santa Barbara, CA, United States; ^3^Kokoro Research Center, Kyoto University, Kyoto, Japan

**Keywords:** culture, interdependence, social rank, social hierarchy, leadership, nonverbal behavior, multilevel analysis, Japan

## Abstract

Previous research has shown that leadership is signaled through nonverbal assertiveness. However, those studies have been mostly conducted in individualistic cultural contexts, such as in the U.S. Here, we suggest that one important strategy for goal attainment in collectivistic cultures is for leaders to self-regulate their behaviors. Thus, contrary to the previous evidence from individualistic cultural contexts, in collectivistic cultural contexts, leaders might suppress nonverbal assertiveness. To test this possibility, we assessed nonverbal behaviors (NVB) of Japanese leaders and members, and how they were evaluated by observers. We recruited Japanese leaders and members of university clubs and video-recorded them while introducing their club. Then, we coded their nonverbal rank signaling behavior. Finally, we asked a new set of naïve observers to watch these video-clips and to judge targets' suitability for being possible club leaders. Results of a multilevel analysis (level 1: individual participants, level 2: clubs) suggested that the more the club culture focused on tasks (rather than relationships), the more likely were leaders (but not members) of those clubs to suppress their nonverbal assertiveness. Naïve observers judged individuals who restrained from emitting nonverbal assertiveness as being more suitable and worthy club leaders. Thus, our findings demonstrate the cultural fit between contextual effects at the collective level (i.e., cultural orientation of a group) and the signaling and perceiving of social ranks at the individual level (i.e., suppression of nonverbal assertiveness). We discuss the importance of studying the cultural fit between the collective reality that people inhabit and people's psychology for future research in cultural psychology.

## Introduction

Social hierarchy is a fundamental dimension organizing interpersonal interactions (Fiske, 1992), because it reduces intragroup conflict (Anderson et al., 2006; Bendersky and Shah, 2012), improves decision-making (Van Vugt et al., 2008), coordinates collective locomotion (Blau, 1964; Hardy and Van Vugt, 2006; Keltner et al., 2008; Willer, 2009), and increases team performance (Halevy et al., 2012; Ronay et al., 2012; Anicich et al., 2015). When clear hierarchies are missing, life becomes difficult (Greer and van Kleef, 2010; Greer et al., 2011). This is true whether we play in teams or work in companies.

To avoid the repeated engagement in costly antagonistic behaviors, social species have developed sophisticated systems of signaling social rank through chemical, visual, auditory, and postural cues (Fernald, 2014). One set of nonverbal cues that signals superior rank is the display of assertiveness and nonverbal dominance (Mazur, 1973; Mazur and Rosa, 1979). For example, taking an expensive body posture, looking another conspecific directly into the eyes, and, in humans, speaking with a loud voice are associated with higher rank (Hall et al., 2005). These signals are readily picked up by conspecifics, who in turn can adjust their behavior depending on where they rank relative to the rank displayed (Tiedens and Fragale, 2003).

Yet, humans are a species rich in sociocultural contexts. Although many studies have examined social hierarchies, they were mainly conducted in individualistic cultural contexts (e.g., North America). Human groups, however, have contrived different culturally subscribed rules and norms for how to best approach and coordinate social living, and how to most appropriately sort group members into higher and lower ranks (Heine, 2012). Thus, currently missing from the discussion of social hierarchy and the scientific investigation of interactions between leaders and followers is a cultural perspective. Particularly, how social hierarchy is constructed, communicated, and sanctioned in interdependent cultural contexts remains largely unknown. Here, we investigate how interdependent cultural contexts shape one important aspect of social hierarchy: the signaling and the perceiving of social rank.

Our theory builds on evolutionary theorizing that leaders serve group functions (Van Vugt, 2006; Price and Van Vugt, 2014), and we combine it with cultural psychology theory suggesting that goals across cultural groups can systematically differ (Markus and Kitayama, 1991; Triandis, 1995). For example, among collectivistic cultures, there is an emphasis on collective goals facilitating interpersonal interdependence (Yamaguchi, 1994) and on leadership styles focusing on the connectedness and social harmony amongst group members (Misumi and Peterson, 1985; Menon et al., 2010). Indeed, there is initial evidence that leaders from collectivistic cultures are psychologically attuned to the needs and well-being of other group members (Zhong et al., 2006; Choi et al., unpublished data).

As a result, we suggest that in contrast to leaders from individualistic cultures, leaders from collectivistic cultures will suppress signals of nonverbal assertiveness. We build on previous theorizing that in interdependent cultural contexts, individuals conform and adjust themselves to others (e.g., Kim and Markus, 1999; Hashimoto and Yamagishi, 2013). In these contexts, strong expressions of assertiveness and dominance endanger the group's social harmony (Hashimoto et al., 2012; Hashimoto and Yamagishi, 2013). Because leaders serve as prototypes for how group members are expected to behave (Hogg, 2001; Hogg et al., 2012), the leader's behavioral self-regulation might especially contribute to the group's social harmony, reducing other group members' assertive behavior and integrating their feelings and opinions. The more successful leaders embody this cultural strategy of behavioral regulation and restrain from expressing nonverbal signals of assertiveness, we reasoned further, the more likely they should be perceived as worthy leaders by other group members.

We test our theory in one particular non-Western collectivistic cultural context of interdependent groups: university student clubs in Japan. Using a thin-slice approach of nonverbal behavior (NVB) (Ambady and Rosenthal, 1992), we investigate the cultural fit between the culture of a given group and their leaders' and members' NVBs, and we also test perceivers' perceptions of leadership worthiness when watching these thin-slice recordings.

### Theory development

According to the functionalist account of social hierarchy, leadership positions are based on the qualities that are most beneficial to the success of a given group (Emerson, 1962; Berger et al., 1972). Unsurprisingly, more prototypical group members are more likely to achieve leadership positions (Hogg, 2001; Hogg et al., 2012). Prototypical leaders are perceived to be more efficient, and they are trusted more, and the better the fit between a target person and the leadership concept held by a given perceiver, the more the perceiver judges this target person as suitable leader (Lord and Maher, 1991).

Importantly, these cognitive representations and leadership schemata can change as a function of the group's goals. For example, while some groups foster leaders who focus their efforts on performance and tasks, other groups foster leaders who focus their efforts on good relationships among group members (Misumi and Peterson, 1985). Indeed, there is a long tradition in leadership research suggesting that individuals can achieve leadership along two pathways: either through demonstrating their individual competence (Blau, 1964; Hollander and Julian, 1969; Lord and Maher, 1991) or through demonstrating their generosity toward group members and through communally-oriented behaviors (Flynn, 2003; Hardy and Van Vugt, 2006; Willer, 2009).

One determinant of group orientation is culture. Cultures represent syndromes of socially shared values and beliefs that characterize a particular society (Triandis, 1995). These worldviews govern the everyday patterns of interactions between members of a given society, including interactions involving social hierarchy. A substantial literature speaks to cultural differences between Western, individualistic cultures, such as the U.S., and East-Asian, collectivistic cultures, such as Japan (Triandis, 1995; Hofstede, 2001).

In Japan, for example, it is of crucial importance to be a part of a group and to maintain harmonious relationships with other group members (Levine and White, 1986; Kitayama and Karasawa, 1995; Kitayama et al., 1997). In fact, individuals from interdependent cultures, such as Japan, define themselves as intrinsically connected to and meaningful through their relationships with others (Markus and Kitayama, 1991). Consequently, in these cultural contexts, people pay more attention to group members (Cohen et al., 2017), and they give priority to collective goals over and above their personal goals (Yamaguchi, 1994). In fact, Japanese who hold a more interdependent self-construal are happier (Uchida et al., 2004; Uchida and Ogihara, 2012). In contrast, pursuing the achievement of individual goals can be harmful for the Japanese well-being, as it can lead to social isolation (Ogihara and Uchida, 2014).

Recent research has started to uncover how cultures shape leadership styles (Hofstede, 2001; House et al., 2004), and there is first evidence that higher social rank is associated with more other- and group-oriented psychological tendencies in Japan (Choi et al., unpublished data). In fact, East Asians' mental representation of leadership is that of the leader standing behind the group, which is an ideal vintage point for group-focused psychological tendencies (Menon et al., 2010). Moreover, priming East Asian participants with social power, a key aspect of leadership, increases their sense of social responsibility (Zhong et al., 2006). It then appears that because groups differ in their shared goals, leadership styles in interdependent cultures, and especially in Japan, might fit their group's focus on social harmony.

The importance of conformity, adjustment to others, and social harmony within interdependent cultural groups is also evident in how their members interact and what NVB they display. For example, Japanese culture puts special emphasis on *omoiyari*, a person's empathetic ability to read another person's feelings and needs, as well as one's readiness to respond to them (Uchida and Kitayama, 2001; Hara, 2006). Thus, strong expressions of assertiveness and dominance are often avoided (Hashimoto et al., 2012; Hashimoto and Yamagishi, 2013). Instead, Japanese tend to exhibit modesty when presenting themselves (Yamagishi et al., 2012), and they tend to engage in more self-criticism (Heine et al., 2000), as to not offend others and prevent disrupting their group's social harmony. We expect that the cultural emphasis on suppressing nonverbal assertiveness will be especially pronounced among prototypes of interdependent cultures, that is their leaders.

Initial support for this idea comes from organizational studies showing that interdependence might be important for the achievement of goals in collectivistic cultures. For example, for efficient goal attainment, middle-class managers in Japan should act as intermediary within the hierarchical network and top manager should follow their middle-class managers advice (Imai et al., 1985; Nonaka, 1988). Of course, too much emphasis on interdependence within organizations will cause severe problems, such as delayed decision-making and low levels of creativity (Numagami et al., 2010). Nevertheless, the importance of interdependence as part of cultural learning about who to select as a leader and how to embody prototypical leadership styles is a crucial aspect of the Japanese culture.

Importantly, cultural differences do not only exist between nations, but are also important characteristics distinguishing different groups within the same nation (Cohen et al., 1996; Uskul et al., 2008; Talhelm et al., 2014). Therefore, we reason that differences in social realities will also be reflected across different groups within a single nation, such as Japan. For example, groups—and especially University clubs - can serve different functions: While some groups focus more on the interpersonal aspect of spending time together and belonging to a social group similar to a family, other groups, such as sport teams, have very clear objectives of achieving goals (i.e., athletic clubs winning championships) (Misumi and Peterson, 1985; Cameron and Quinn, 2006).

As a result, we expect that the cultural fit between leaders' NVB and their group's culture would be especially strong for those groups that focus on achieving goals, because leadership would be required for in-group cooperation and collective action (Van Vugt, 2006; Van Vugt et al., 2008). In other words, to the extent that it is the leader's role to facilitate the attainment of goals within a club, restraining from expressing nonverbal dominance might be especially important. Group members picking up such suppression of nonverbal assertiveness, we predict further, will evaluate these signalers as more suitable and worthy leaders. Thus, another contribution of our research is to demonstrate that even within interdependent nations, there exist important group-level contextual variations, whereby leaders of task-oriented groups restrain even more from emitting nonverbal assertiveness signals.

In the present study, we videotaped members and leaders from Japanese university clubs while they talked about their club. Then, we coded targets' NVBs for cues of assertiveness or the suppression thereof. Naïve Japanese observers estimated targets' suitability as possible club leader. Both coding and estimation were conducted based on 30 s thin-slices of these recordings. Hypothesis 1 holds that Japanese leaders are more likely to suppress signals of nonverbal assertiveness, and this suppression of nonverbal assertiveness should be especially pronounced when their group's task orientation is high. Hypothesis 2 holds that the reduced emission of nonverbal signals of assertiveness should be perceived as signal of greater leadership ability and worthiness in Japanese culture.

## Materials and methods

The procedures used in this work were in accordance with the Japanese Psychological Association ethical guidelines and approved by the Kokoro Research Center at Kyoto University. All participants gave their informed consent and were debriefed and informed about the true purpose of the research immediately after the experiment.

### Recording target videos

#### Participants

We recruited 40 undergraduate students from three universities in Kyoto and Hyogo prefecture (12 females; *M*_age_ = 20.7; *SD*_age_ = 0.81) through flyer distribution and referrals. All participants belonged to university clubs. We recruited one leader (e.g., president) and one member from each university club, but all students participated in this study individually. Participants were recruited such that within each pair, they were of the same sex and same school year^1^. One club member was also the leader of a different club, so that we excluded this pair from analyses. Therefore, final analyses were based on 19 leader-member pairs.

#### Materials and procedure

We video-recorded participants while they gave a short oral introduction to their university club. Participants were given 5 min to prepare for how they wanted to introduce their club. They then spoke for approximately 2 min about their club, while we video-recorded them in a standardized manner. First, we videotaped their whole-body image without targets talking for around 5 s. Then, we adjusted the camera position to capture targets' upper body and filmed their oral introduction. Once the video-recording was completed, targets filled in a questionnaire including demographic questions.

As part of this questionnaire, and to assess how much their club culture was focused on goal attainment, participants answered one item taken from the Organizational Culture Assessment Instrument (Cameron and Quinn, 2006); “My club is very results-oriented. A major concern is with getting the job done. People are very competitive and achievement-oriented.” Participants answered this item on a 9-point scale ranging from “1-not at all” to “9-very much.”

We edited each 2-min video into a 30 s thin-slice clip. Each thin-slice clip consisted of three parts; a whole-body shot (around 4 s; showing participants' whole body without them talking), an earlier speech shot (around 13 s from mostly the first minute of their speech) and a later speech shot (around 13 s from mostly the second minute of their speech). We extracted speech shots from two different time points as to account for the possibility that targets might have been nervous at beginning of their speech, but might have been more relaxed and fluent as they kept talking.

### Coding nonverbal behavior

#### Participants

Two authors (one Japanese male and one non-Japanese male) and one additional Japanese female unfamiliar with the research hypotheses coded the 40 edited videos.

#### Materials and procedure

We created a coding schema of NVB based on a seminal meta-analysis by Hall et al. (2005), who reviewed studies investigating the relationship between social rank and NVB rank signals. The coders watched the 30 s thin-slice video-clips with the sound on and rated each NVB category based on the coding schema on a scale ranging from 1 to 5 (see Table 1 for the coding schema and inter-rater reliability of each category).

**Table 1:** Nonverbal behavior categories and inter-rater reliability.

**Category**	**Definition**	**Scale**		**Cronbach's alpha**
		**1** ~	**5**	
Smiling	How frequently and intensely the target smiles	No smiling	Smiles a lot	0.92
Eye contact	How long the target looks at the camera	Avoiding the camera	Direct gazing into the camera	0.87
Nodding	How frequently the target nods	No nodding	A lot of nodding	0.86
Arm position	Where the target's arms are placed	Mostly in front of body	Mostly behind the body	0.99
Nervous hands	How frequently the target touches himself/herself	No nervous hand movements	A lot of nervous hand movements	0.90
Explaining gestures	How frequently and intensely the target gestures	No gesturing	A lot of gesturing	0.95
Postural relaxation	To what extent the target is relaxed (particularly focusing on his/her shoulders)	Not relaxed	Relaxed	0.76
Body shifting	How frequently and intensely the target moves his/her torso	Still and erect	Moves a lot	0.85

### Judging leadership suitability

#### Participants

We recruited a new sample of 38 undergraduate students from Kyoto University (19 females; *M*_age_ = 20.3; *SD*_age_ = 1.50) to watch the video-clips and evaluate the targets. We excluded one participant who was partly socialized within another cultural context. Therefore, final analyses were based on 37participants.

#### Material and procedure

Judges watched the 30 s thin-slice video-clips and assessed how much they thought the targets were suited as a leader of a university club in general on a 6-point scale ranging from “1-not at all” to “6-very much.” Then, they answered whether they knew the target. Participants viewed 20 targets, one video-clip at a time, with thin-slice video-clips pseudo-randomly selected from the 40 video-clips (video-clips were presented with equal frequencies across the experiment) and presented in random order. We showed the video-clips at a resolution of 960 pixels × 540 pixels with the sound muted as to have participants assess the perceived leadership worthiness based on the NVB alone, but not based on targets' speech content. We excluded trials from analyses, in which participants reported that they knew thetarget.

## Results

### Computing nonverbal behavior scores

Table 2 shows descriptive statistics for all NVB categories. Because some NVB scores were skewed, we conducted a categorical exploratory factor analysis with median scores of NVB instead of conventional exploratory factor analysis with mean scores. Specifically, we used WLSMV estimator (weighted least squares with mean and variance adjustment) and geomin in Mplus Version 8 (Muthén and Muthén, 1998–2017). When including all NVB categories, 3 factors yielded Eigenvalues larger than 1, but only the two-factor model yielded adequate model fit [χ${}_{\left(13\right)}^{2}$ = 13.12, *p* = 0.44; RMSEA = 0.02; SRMR = 0.08]. In this model, smiling and nodding had low communality (0.13 and 0.13 respectively), so that we re-ran the analysis without these two NVB categories. This time, 2 factors yielded Eigenvalues larger than 1 and only the two-factor model provided adequate model fit [χ${}_{\left(4\right)}^{2}$ = 1.30, *p* = 0.86; RMSEA = 0.00; SRMR = 0.03]. Because this model also showed interpretable factor loadings and acceptable communality for all NVB categories (Table 3), we computed the corresponding factor scores. Factor 1 consisted of a NVB style, where participants kept arms behind the body, did not fiddle, and showed a relaxed posture (particularly in their shoulders). Because this style was characterized by calm, restrained and suppressed NVB, we called this factor score “nonverbal restraint.” In contrast, Factor 2 consisted of a more expressive NVB style including gesturing, bodily shifting, and having one's gaze wonder around. We called this factor score “nonverbal expression.” These two factor scores were negatively correlated with each other [*r* = − 0.52, *t*_(36)_ = −3.63, *p* = 0.001].

**Table 2:** Descriptive statistics of each nonverbal behavior category.

**Category**	***M***	**SD**	**Spearman's rank-order correlation**							
			**1**	**2**	**3**	**4**	**5**	**6**	**7**	**8**
1. Smiling	2.55	1.27	-	−0.21	0.30	−0.07	0.22	0.22	−0.17	0.26
2. Eye contact	3.38	1.05		-	−0.05	0.26	−0.36	−0.37	0.08	−0.49
3. Nodding	2.77	0.98			-	−0.14	0.27	−0.07	−0.21	0.06
4. Arm position	2.58	1.66				-	−0.59	−0.38	0.25	−0.19
5. Nervous hands	2.10	1.19					-	0.40	−0.29	0.21
6. Explaining gestures	1.61	1.03						-	−0.09	0.25
7. Postural relaxation	3.30	0.83							-	0.07
8. Body shifting	3.00	0.96								-

*These statistics are based on mean scores (not median scores)*.

**Table 3:** Factor analysis of nonverbal behavior categories.

**Category**	**Factor 1**	**Factor 2**	**Communality**
Nervous hands	−0.91	0.00	0.84
Arm position	0.58	−0.27	0.54
Postural relaxation	0.54	0.11	0.26
Explaining gestures	0.00	0.68	0.47
Body shifting	0.25	0.68	0.39
Eye contact	0.10	−0.64	0.47
Eigenvalue	2.53	1.37	

### Signaling social rank through nonverbal behavior

To test our key hypothesis whether club leaders compared to club members would signal their superior social rank and whether this tendency was enhanced within task-oriented club cultures, we conducted a hierarchical linear model with NVB as the dependent variable (see Table 4 for descriptive statistics of variables used in the analysis):

**Table 4:** Descriptive statistics of variables used in hierarchical linear modeling.

**Variable**	***M***	**SD**	**Pearson correlation coefficient**				
			**1**	**2**	**3**	**4**	**5**
1. Social rank	0.00	0.50	-	0.07	0	0.02	0.02
2. Task-oriented club culture	5.05	2.28		-	−0.11	0.28	−0.14
3. Sex	0.32	0.46			-	−0.39	0.06
4. Nonverbal restraint	−0.02	0.82				-	−0.52
5. Nonverbal expression	0.00	0.80					-

*Social rank was a dichotomous variable with −0.5 = club member and +0.5 = club leader. Sex was also a dichotomous variable with 0 = male and 1 = female*.

Individual Level:

$$\begin{array}{l} NVB_{ij}=\beta_{0j}+\beta_{1j}{\left(Social\;Rank\right)}_{ij}+\beta_{2}(Task-Oriented\\ \;\;Club\;Culture_{individual}{)}_{ij}+\beta_{3}(Social\;Rank\\ \;\times \;Task-Oriented\;Club\;Culture_{individual}{)}_{ij}+r_{ij} \end{array}$$

Group Level:

$$\begin{array}{l} \beta_{0j}=\gamma_{00}+\gamma_{01}{\left(Task-Oriented\;Club\;Culture_{group}\right)}_{j}\\ \;+\gamma_{02}{\left(Sex\right)}_{j}+\mu_{0j}\\ \beta_{1j}=\gamma_{10}+\gamma_{11}{\left(Task-oriented\;Club\;Culture_{group}\right)}_{j}\\ \;+\gamma_{12}{\left(Sex\right)}_{j}+\mu_{1j} \end{array}$$

The *i* subscript refers to the individual-level observations. The *j* subscript refers to group-level observations (i.e., university club). We decomposed task-oriented club culture by calculating group means (as a group-level variable; *task-oriented club culture*_*group*_ in the equations above) and group-mean centered scores (as an individual-level variable; *task-oriented club culture*_*individual*_ in the equations above). With Mplus Version 8 (Muthén and Muthén, 1998–2017), we tested this model applying a Bayesian statistical framework (using non-informative priors and Markov chain Monte Carlo methods) to avoid non-convergence problems, which tend to occur especially when estimating relatively complex multivariate models with small samples in maximal likelihood method (e.g., Ozechowski, 2014). Twenty thousand simulated draws from the posterior were obtained for each parameter. The simulated draws were preceded by 10,000 burn-in draws, which were discarded from the analysis. To reduce temporal autocorrelation among the draws, the MCMC chain was thinned by including only every 5th draw, yielding 4,000 simulated posterior observations. We assessed convergence by using the Gelman-Rubin convergence criterion based on the potential scale reduction factor for each parameter (Gelman and Rubin, 1992).

#### The effects of social rank and task-oriented club culture on nonverbal restraint

To determine whether the group-level (clubs) variance of a given variable was to be considered, we checked Intra-Class-Correlations (ICC) of task-oriented club culture and nonverbal restraint. Because both variables had high ICC values (0.60 for task-oriented club culture, 0.17 for nonverbal restraint), we decomposed these variables into two levels; individual-level (participants) and group-level (clubs). As for task-oriented club culture, we decomposed it by calculating group means (as a group-level variable) and group-mean centered scores (as an individual-level variable). Moreover, we included social rank (−0.5 = Member, +0.5 = Leader) as an individual-level variable and participants' sex (0 = Male, 1 = Female) as a group-level variable because it was matched within pairs.

**Figure 1:**
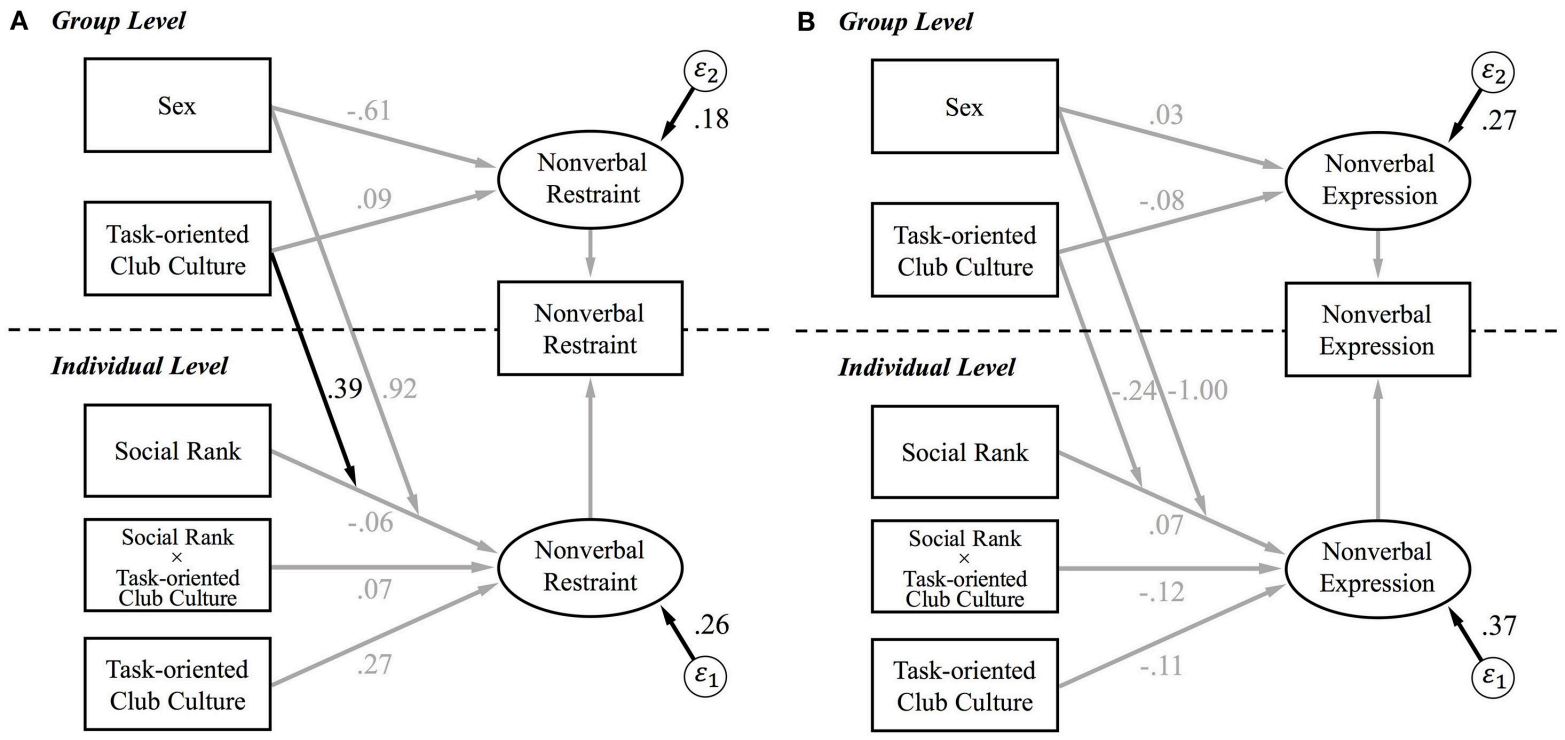
Hierarchical linear modeling results predicting nonverbal behavior as a function of social rank and task-oriented club culture. Black lines represent significant paths, and gray lines represent nonsignificant paths. Social Rank was a dichotomous variable with −0.5 = club member and +0.5 = club leader. Sex was also a dichotomous variable where 0 = male and 1 = female. Task-oriented club culture at both levels and sex were centered. **(A)** The cross-level interaction of individual-level social rank and group-level task-oriented club culture on nonverbal suppression was significant. Estimated residual variance of the random slope was 0.76. **(B)** The cross-level interaction of individual-level social rank and group-level task-oriented club culture on nonverbal expression was not significant. Estimated residual variance of the random slope was 1.00.

Figure 1A shows the hierarchical linear model (DIC = 58.66). At the individual-level, neither participants' social rank, nor task-oriented club culture, nor their interaction term predicted nonverbal restraint (social rank: *b* = −0.06, 95% credible interval [−0.62, 0.51]; task-oriented club culture: *b* = 0.27, 95% credible interval [−0.03, 0.57]; interaction between social rank and task-oriented club culture: *b* = 0.07, 95% credible interval [−0.51, 0.64]). As predicted, we observed a cross-level interaction between participants' social rank at the individual level and task-oriented club culture at the group-level (*b* = 0.39, 95% credible interval [0.09, 0.69]). The stronger the task-oriented club culture was, the more leaders compared to members suppressed nonverbal assertiveness. Simple slope analysis for the cross-level interaction missed conventional levels of significance at +1SD and−1SD of the group-level task-oriented club culture (+1SD: *b* = 0.74, 95% credible interval [−0.06, 1.53]; −1SD: *b* = −0.85, 95% credible interval [−1.70, 0.00]), but yielded significantly different results from zero at the maximum and the minimum values (max: *b* = 1.10, 95% credible interval [0.08, 2.10]; min: *b* = −1.25, 95% credible interval [−2.35, −0.16]). Thus, the cross-level interaction seemed especially pronounced for more extreme values of task-orientation. This finding partially confirms Hypothesis 1.

#### The effects of social rank and task-oriented club culture on nonverbal expression

To determine whether the group-level (clubs) variance of a given variable was to be considered, we checked ICC of task-oriented club culture and nonverbal expression. Because both variables had high ICC values (0.60 for task-oriented club culture, 0.16 for nonverbal expression), we decomposed these variables into two levels; individual-level (participants) and group-level (clubs). Again, we decomposed task-oriented club culture by calculating group means (for a group-level variable) and group-mean centered scores (for an individual-level variable). Moreover, we included social rank (−0.5 = Member, +0.5 = Leader) as an individual-level variable and participants' sex (0 = Male, 1 = Female) as a group-level variable because participants' sex was matched within pairs.

Figure 1B shows the hierarchical linear model (DIC = 72.31). Importantly, a significant cross-level interaction between participants' social rank at the individual-level and task-oriented club culture at the group-level did not emerge for nonverbal expression (*b* = −0.24, 95% credible interval [−0.59, 0.11]).

### Perceptions of suitable leadership

Finally, we tested whether more restrained or expressed NVB would be perceived as more suitable for leadership using lmerTest package in R (Kuznetsova et al., 2017). We regressed the perceived-leadership-worthiness score on nonverbal restraint and nonverbal expression separately, controlling for targets' sex. We also specified a random intercept of targets and a random intercept and slope of judges in these analyses. As we expected, the main effect of nonverbal restraint on the perceived-leadership-worthiness score was marginally significant (*b* = 0.25, *SE* = 0.13, *p* = 0.07). The main effect of nonverbal expression on the perceived-leadership-worthiness score, however, was not significant (*b* = −0.09, *SE* = 0.13, *p* = 0.47). That is, in support of Hypothesis 2, naïve Japanese participants judged targets who restrained from emitting nonverbal assertiveness as somewhat more suitable and worthy of being possible club leaders.

## Discussion

In the present research, we set out to investigate the nonverbal signaling of leaders and followers within interdependent groups. Specifically, we focused on Japanese leaders and members of university clubs. Consistent with our predictions, we found that when the Japanese university club culture focused on goal attainment, leaders' NVB was characterized by restraining from assertive nonverbal signals. Naïve Japanese observers judged targets who showed nonverbal restraint as more suitable and worthy of being possible club leaders. Thus, our findings are consistent with functional approaches to leadership, which suggest that culture-specific prototypical leadership styles are beneficial to group outcomes. Moreover, our research provides an important case study for how the cultural orientation of a group and the NVB of its leader match and fit the group-level cultural context.

Our research makes a significant contribution to the leadership literature across cultures. For example, we find that in contrast to Western leaders who focus on the expression of nonverbal assertiveness (Mazur and Rosa, 1979), East Asian leaders focus more on the suppression of nonverbal assertiveness. Such suppression of nonverbal assertiveness seems functional within a cultural context that is geared towards interpersonal connectedness and social harmony. Indeed, previous research has shown that East Asian leaders are mentally represented as standing behind the group, where they can better focus on other group members' needs (Menon et al., 2010). East Asians but not Westerners primed with social power activate more responsibility-related mental concepts (Zhong et al., 2006), and Japanese leaders show more other-oriented psychological tendencies than their American counterparts (Choi et al., unpublished data). Here, we demonstrate that leaders in interdependent groups also adapt their NVB to fit the interdependent cultural orientation.

Cultures do not only differ in how their leaders maintain and signal rank, but also in who they select into positions of leadership. For example, Torelli et al. (2014) have shown that in more collectivistic groups, leaders ought not only to be competent but also warm, and they display warmth and competence equally frequent. Other research has shown that ascribed status characteristics, such as coming from a privileged family background, are important for occupying high rank positions in high power distance cultures (Gobel et al., unpublished data). These authors also showed that when the social reality was described as one of rigid hierarchies compared to when social reality was described as one of malleable hierarchies, participants placed more emphasis on ascribed status characteristics when selecting future team leaders. Thus, the social reality that people encounter as part of their everyday culture plays an important role in who they select into positions of leadership.

In addition, our research contributes to the literature on NVB. Specifically, our factor analysis yielded two distinct factors of NVB, nonverbal restraint and nonverbal assertiveness. This suggests that nonverbal restraint could be a distinct nonverbal rank-signaling style. Indeed, it is conceivable that nonverbal restraint might not only convey the absence of nonverbal assertiveness, but that it may also reflect additional desirable leadership traits (e.g., showing confidence and reliability). Our study provides a glimpse into this possibility and opens a new pathway for future NVB studies wishing to further explore the meaning of nonverbal restraint as rank signal.

Our research also makes a significant contribution to the cultural psychology literature demonstrating the importance of studying the influence of shared social realities at the collective level. For example, collective levels of income predict independent cultural orientations among Chinese (Takemura et al., 2016), state-level collectivism predicts psychological resilience when facing threat among Americans (Kim et al., 2016), and residential mobility at the collective level is a stronger moderator of social cooperation than residential mobility at the individual level (Takemura, Fukushima, and Uchida, unpublished data). Similarly, in social hierarchies, the shared social reality plays an important role in determining an individual's social rank. For example, Li et al. (2016) showed the importance of social contexts, similar to the distinction between task-orientated and relationship-orientated groups in our study, on status attainment within a given group. We add to this literature, demonstrating the cultural fit between a group's cultural orientation to focus on the achievement of goals at the collective level and the leaders' suppression of nonverbal assertiveness signals at the individual level.

It is important to note that our research has strengths and limitations. First, we applied hierarchical linear modeling to gauge the effects of shared cultural orientation at the collective level (i.e., university club culture) on NVB at the individual level (i.e., leader's nonverbal assertiveness suppression). While this analytic approach yielded important new insights into cultural fit, it should be acknowledged that our sample was at the lower end of typical sample sizes employing such analytical approaches. Thus, future efforts could benefit from sampling larger number of clusters (i.e., groups).

Our research focused on real-world leaders from interdependent groups, and we assessed their actual NVB. While this data provides important new insights into the functioning of informal social hierarchies, more work is needed to understand the nature of rank signaling within formal social hierarchies in interdependent group contexts. One possibility is that when social hierarchy is defined through formal ranks (e.g., in companies), East Asian leaders might also show more nonverbal assertiveness. For example, previous research showed that the expression of anger has a distinct function in formal Japanese hierarchies (Park et al., 2013; Kitayama et al., 2015). Another possibility is that nonverbal assertiveness is expressed in context-dependent manner, such that leaders both suppress and express nonverbal assertiveness displaying a mixed leadership style (Misumi and Peterson, 1985). Future research is needed to study leaders' NVB in formal social hierarchies and in interdependent group contexts.

In the present study, we chose to show videos of leaders' NVB without sound. This was done in order to avoid naïve perceivers deducing targets' social rank based on the speech content. Yet, this decision deprived observers from important cues of social rank such as targets' pitch of voice, their speech fluency and loudness of their voice. While it is interesting that our results show a meaningful association of suppressing nonverbal assertiveness and perceptions of being a suitable and worthy leader, future research could include the sound of the videos to test how these additional cues of NVB would affect participants' judgments.

Finally, it should be mentioned that the current study did not directly compare across independent and interdependent groups. Thus, while previous research is suggestive of the fact that cultures significantly differ in the extent to which they signal social rank (e.g., Gobel et al., 2015, 2017), it remains unknown how university club leaders and members from more individualistic cultural contexts, such as the U.S., signal their social rank. While our theory predicts that they should express more assertiveness to advance their group's goals, this remains an empirical question, which we are currently exploring.

In sum, we demonstrate that among a sample of Japanese interdependent university clubs, leaders more than members suppress emitting nonverbal assertiveness, when the collective understanding of the group's purpose is to achieve goals. Therein, our research makes an important contribution to the study of cultural fit between contextual effects at the collective level (i.e., group) and behavior at the individual level. We suggest that such cultural fit between the social reality that people inhabit and their individual-level psychology might be the next frontier in cultural psychology research.

## Ethics statement

This study was carried out in accordance with the recommendations for Ethical Principles of Psychologists. All subjects gave written informed consent in accordance with the Declaration of Helsinki. The protocol followed the ethical guidelines of the Japanese Psychological Association.

## Author contributions

All authors contributed to the study design. Data collection and the data analysis were performed by AI. AI and MG drafted the manuscript, and YU provided critical revisions. All authors approved the final version of the manuscript for submission.

### Conflict of interest statement

The authors declare that the research was conducted in the absence of any commercial or financial relationships that could be construed as a potential conflict of interest.

## Footnotes

^1^Although one leader-member pair was not in the same school year, we maintained their data and included them in analyses. Excluding this pair does not change the general pattern of results.
